# Prevalence of HIV and sexually transmitted infections among clients of female sex workers in Karnataka, India: a cross-sectional study

**DOI:** 10.1186/1471-2458-11-S6-S4

**Published:** 2011-12-29

**Authors:** Souradet Y Shaw, Kathleen N Deering, Sushena Reza-Paul, Shajy Isac, Banadakoppa M Ramesh, Reynold Washington, Stephen Moses, James F Blanchard

**Affiliations:** 1 Centre for Global Public Health, Department of Community Health Sciences, University of Manitoba; Winnipeg, Canada; 2 School of Population and Public Health, University of British Columbia; Vancouver, Canada; 3 Karnataka Health Promotion Trust; Bangalore, India; 4 Department of Medical Microbiology, University of Manitoba; Winnipeg, Canada

## Abstract

**Background:**

Studies have demonstrated the significance of commercial sex work in the ongoing transmission of HIV and other sexually transmitted infections (STIs) in India. Clients of female sex workers (FSWs) are thought to be an important bridging population for HIV/STIs. However, there is a lack of information on basic characteristics of sex work clients. This study sought to describe the prevalence of HIV and other STIs, as well as examine the determinants of these pathogens among a sample of clients in south India.

**Methods:**

Data were from a cross-sectional biological and behavioural survey of FSW clients from six districts in Karnataka State, India. The prevalence of HIV, syphilis, herpes simplex virus type 2 (HSV-2), chlamydia (CT) and gonorrhoea (NG) among clients was examined. Multivariable logistic regression models were used to analyse the socio-demographic, sexual behaviour and sex-work related characteristics related to the prevalence of each pathogen. Sampling weights and appropriate survey methods were utilized in regression models to account for complex sampling design.

**Results:**

The total sample size was 2,745. The average age of clients was 30.4 (SE:0.3). Across the total sample, the prevalence of HIV, HSV-2, syphilis and CT/NG was 5.6%, 28.4%, 3.6% and 2.2%, respectively. The prevalence of HIV/STIs varied substantially across districts, reaching statistical significance for HIV (p<.0001) and CT/NG (p=.005). In multivariable models, duration of paying for commercial sex was associated with increased risk for HIV and HSV-2 (AOR: 1.1; 95%CI: 1.0-1.1, p<.0001). Clients with brothels as a main FSW solicitation site were associated with increased risk of HIV (AOR: 2.4; 95%CI: 1.2-4.7, p=.001), while those frequenting lodges were at increased risk for CT/NG (AOR: 6.3; 95%CI: 1.9-20.6, p=.03). Examining co-infections, clients with HSV-2 infections were at substantially higher risk of being HIV-positive (AOR: 10.4; 95%CI: 6.1-17.7, p<.0001).

**Conclusions:**

This study fills in important gaps in knowledge regarding clients in southern India. The strong association between HIV and HSV-2 infections highlights the complications in designing effective prevention, intervention and management programs of this well-hidden population.

## Background

Reducing the transmission of HIV and other sexually transmitted infections (STIs) remains a public health priority in India [1]. As in other parts of Asia, although HIV epidemiology is thought to be fundamentally complex [2], with elevated risk among several ‘high-risk’ sub-populations, such as men who have sex with men (MSM) [3-8], the predominant driver of HIV transmission in India remains sex work [8-11].

Within the context of sex work, it has been recognized that considerable variation exists in the relationships between female sex workers (FSWs) and their clients, and the behaviours and interactions between the two groups, resulting in substantial heterogeneity in the nature of risk for HIV transmission [12-16]. Ultimately, the differences in the social and structural organisation of sex work are manifested by variations in HIV and STI prevalence among different FSW populations [15,16].

Although high prevalence of HIV/STIs and risk behaviours associated with ongoing transmission has been observed in clients globally [17-20], with a handful of exceptions [21,22], very little is known about the prevalence and correlates of HIV/STIs among clients in India. One previous study of clients from 12 districts in India found that HIV prevalence ranged from 2% to 10%, depending on district. Although no statistically significant associations were found between HIV and factors included in their analysis, the authors found that two measures of high risk behaviour, volume of sex acts and inconsistent condom use, were positively associated with older age and having a mix of both commercial and non-commercial partners [21]. These findings align with the notion of clients as an important bridging population, with the potential to further the transmission of HIV and other STIs to both commercial and non-commercial partners, described in other localities [2,11,23,24].

Given the high rates, by Indian men, of both commercial sex partnerships and inconsistent condom use [25], and the lack of access to basic HIV prevention services [26], there is a need to describe the epidemiology of HIV and other STIs in clients from India. This study therefore sought to characterise the prevalence, and factors related to HIV and other STIs among a sample of clients. As developing interventions that are tailored for FSWs and their clients is thought to be pivotal in reducing HIV incidence further [27], the results of this study can be used to inform client-focussed prevention programmes.

## Methods

### Study design and sampling

Data were obtained from a cross-sectional behavioural and biological survey of clients in six districts (i.e. sub-state administrative areas, including Bagalkot, Bangalore, Belgaum, Bellary, Mysore and Shimoga) in Karnataka State, southern India. Districts were chosen purposively, based on size of high risk populations (in this case FSWs, with the rationale being large FSW populations and client populations are correlated) and the socio-cultural regions of Karnataka [16]. Clients were recruited in 2008 through a multistage cluster sampling technique, similar to that described in Subramanian et al. [21]. Briefly, selection of solicitation sites occurred in the first stage of sampling, and selection of clients in the second. Depending on place of solicitation, all clients were identified by FSWs, madams, brothel owners or through visible clues suggestive of seeking sex workers. Field supervisors would then approach and recruit clients until a target number of clients was reached. For the purposes of this study, men were included in the study if they were between 18 and 60 years of age, and reported exchanging money for sex with FSWs in the past month. Clients were truncated at age 60 to focus on those that were thought to be more sexually active. Overall response rate was 82%, with lowest response rates observed in Bangalore (70%), and highest in Bagalkot (90%).

### Survey organization and methods

Men were assigned study numbers for de-identification, and interviewed individually through a structured questionnaire administered face-to-face by trained workers in the local language. As in previous studies, biological data for HIV, herpes simplex virus type 2 (HSV-2), syphilis, and chlamydial (CT) and gonorrhoeal (NG) infections were gathered using blood and urine samples [21]. Blood was tested for HIV, syphilis and HSV-2 using standard serological tests, and urine was tested with a nucleic acid amplification assay (Gen-Probe Aptima) for the detection of CT and NG. Although surveys and the results of specimens were made anonymous at collection, a mechanism was in place to notify, by study number, those who were positive for syphilis. These clients would be asked to attend a clinic for treatment. Other STIs were treated syndromically. HIV results were not notified individually, but all individuals were given vouchers for free HIV testing and counseling at government testing centres. Written, informed consent was obtained for participation in the study and the institutional review boards at the University of Manitoba in Winnipeg, Canada and St. John’s University in Bangalore, India approved the study.

### Measures

Respondents were compared on socio-demographic, sexual behaviour and sex-work related characteristics. Age, literacy levels, district of residence, and occupation were included as socio-demographic variables. Age at first vaginal intercourse, condom use patterns with intimate partners (i.e. regular, non-commercial partnerships) and reported anal sex with MSM or *hijras* (i.e., transgendered MSM) were used as sexual behaviour variables. Age at first paid sex, duration of paid sex, usual place of solicitation of FSWs, number of different FSWs in the last 6 months (total, and the proportion of regular and occasional FSWs), and condom use patterns with regular (i.e. FSWs whom clients seen more than once) and occasional (i.e., non-regular or casual) FSWs were used as sex work-related variables. For condom use, the following questions were asked, depending on type of partner (“Partner Type X”). First, condom use at last sex was assessed by the question: “Was a condom used the last time you had sexual intercourse with *Partner Type X*?” Second, clients were asked “In general, how often do you use condoms with *Partner Type X*?” Clients were allowed to choose from the following: Everytime; Most of the time; Sometimes; and Never. This variable was then made dichotomous, with those answering “Never” categorized into one group, and all other remaining answers into the other group.

### Statistical analysis

In bivariate analyses, chi-square tests were used to assess associations between socio-demographic, sexual behaviour and sex work-related characteristics and each pathogen under investigation. Because of the use of multiple comparisons, and as each variable was essentially tested four separate times in bivariate analyses, an association was only considered significant at the p<.0125 level (.05/4). Two sets of multivariable logistic regression analyses were conducted. First, in order to assess factors independently related to the prevalence of each specific pathogen, separate multivariable logistic regression models were constructed with each pathogen used as the outcome variable. Second, the presence of HIV was used as the outcome variable only, with all other pathogens used as predictor variables while simultaneously adjusting for the same factors in the first set of models. Because of the potential of the large sample size to detect not necessarily meaningful statistical associations, and as the main purpose of the analyses was to make comparisons *between* pathogens, the same independent variables were used for each pathogen, and for the coinfection comparisons. Thus, *a priori*, and based on a conceptual understanding of sex work in India, duration of paid sex, ability to read or write, marital status, number of FSW contacts in the last 6 months, district of residence, place of solicitation and occupation were adjusted for in multivariable models. Age and duration of paid sex were highly correlated (r^2^=0.88). Given the impact of collinear variables on the precision of estimation [28], only duration of paid sex was included in multivariable analyses. As a sensitivity analysis, all multivariable models were repeated with age in place of duration. Sampling weights were utilized in multiple regression models to account for the complex sampling design, using survey methods in Stata 11 [29]. Multicollinearity in multivariable models was assessed using the variance inflation factor (VIF) and tolerance statistics, corrected for the survey methods employed [30].

## Results

A total of 2757 clients were included in the study. Additional file 1 includes the socio-demographic, sexual behaviour and sex-work related characteristics of the study sample. The average age of clients was 30 years, with 46% over the age of 30 at the time of the interview. The majority of respondents were married (62%), with just under half belonging in the “Other (including Labourer)” category for occupation. Respondents from Bangalore comprised approximately 31% of the sample. The majority (66%) of respondents reported having a regular non-commercial intimate partner (including spouse), with condom usage being low among intimate partners. Approximately the same proportion of respondents reported ever having anal sex with their intimate partners (5%) as having anal sex with another man or hijra in the last 6 months (6%).

Mean age of first paid sex was 22 years, with the average duration of visiting FSWs being 9 years. Respondents were most likely to report the FSWs’ home (48%) and public places (36%) as the places most frequently used to solicit FSWs. Respondents reported visiting an average of 4.3 different FSWs in the 6 months prior to their interview, with approximately 85% having had at least one contact with an occasional FSW and 42% reporting at least one contact with a regular FSW. Although practically speaking, the difference is minimal, condom use was slightly higher with occasional FSWs, with 67% of respondents reporting condom use at last sex with occasional FSWs, compared to 60% with regular FSWs.

Of the 2757 clients included in the study, 2745, 2610, 2613 and 2736 were tested for HIV, HSV-2, syphilis and CT/NG, respectively. HSV-2 was the most prevalent infection, at 28%, followed by HIV at 6%. Active syphilis was detected in 4% of the study sample who were tested for syphilis. The prevalence of CT and NG were 2% and 1%, respectively, with a prevalence of either infection being 2%.

### Bivariate analyses

Additional file 2 shows the prevalence of HIV and other pathogens by socio-demographic, sexual behaviour and sex-work related characteristics; results from chi-square tests of association from bivariate analyses investigating the association between each pathogen and characteristics of interest are displayed. Age was significantly associated with HIV (p=.002) and HSV-2 (p<.0001). Similarly, marital status was significantly associated with HIV (p<.0001) and HSV-2 (p<.0001). Occupation was associated with HIV only (p<.0001), with clients employed in the service industry having the highest prevalence (9%). Literacy level was significantly associated with all four pathogens, with HIV (p<.0001), HSV-2 (p<.0001) and syphilis (p=.0006) less prevalent in those that could read or write. Bagalkot district had the highest HIV and HSV-2 prevalence, at 14% and 36%, respectively, while syphilis prevalence was highest in Bellary (6%) and CT/NG highest in Mysore (5%). Only HIV (p<.0001) and CT/NG (p=.005) differed significantly across districts. Having an intimate partner was associated with higher levels of HSV-2 (p<.0001); HSV-2 prevalence among clients with intimate partners was 34%, while it was 18% among those without intimate partners. Condom use at last sex with an intimate partner was associated with CT/NG prevalence (p=.008).

Duration of visiting FSWs was positively associated with the prevalence of both HIV (p=.001) and HSV-2 (p<.0001). For example, HIV prevalence was 2% among respondents who had been visiting FSWs for a year or less and 8% among those with 10 or more years visiting FSWs. Place of solicitation was associated with HIV (p=.003). Clients who visited brothels had the highest HIV prevalence (9%), while those visiting lodges had the highest CT/NG prevalence (7%). Condom use was associated with the prevalence of HSV-2. Prevalence was higher among clients reporting never using condoms with their occasional (33% vs. 26%, p=.003) FSW partners.

### Co-infections

Additional file 2 also shows the degree to which pathogens were related. HIV prevalence was positively and significantly related to syphilis and HSV-2 prevalence. For example, 21% of clients with syphilis were also HIV-positive, compared to 5% of clients that were not infected with syphilis (p<.0001). Similarly, elevated prevalence of HIV was seen amongst respondents who were HSV-2-positive; 16% of HSV-2-positive respondents were HIV-positive, compared to 2% of those that were not HSV-2-positive (p<.0001). Correspondingly, the prevalence of HSV-2 in those respondents who were HIV-positive was 79%, compared to 25% in HIV-negative respondents.

### Multivariable models

Table 1 shows the adjusted odds ratios (AOR) and 95% confidence intervals (95%CI) from multivariable logistic regression models examining factors associated with each individual pathogen. For HIV, respondents with a longer duration of visiting FSWs were at higher odds of being HIV-positive (AOR: 1.1; 95%CI: 1.0-1.1, p<.0001), adjusted for all other factors in the model. Respondents from Bagalkot (AOR: 2.9; 95%CI: 1.7-5.1, p<.0001), those reporting “service industry” as their primary occupation (AOR: 2.8; 95%CI: 1.5-5.2, p<.01), and those soliciting FSWs in brothels (AOR: 2.4; 95%CI: 1.2, 4.7, p<.01) were all at increased risk of being HIV-positive. Respondents from Bangalore (AOR: 0.4; 95%CI: 0.2-0.7, p<.01), relative to other districts were at decreased risk of HIV. For HSV-2 a longer duration of visiting FSWs (AOR: 1.1; 95%CI: 1.1-1.1, p<.001), and being from Bagalkot, relative to other districts (AOR: 1.6; 95%CI: 1.2-2.3, p<.01) were all associated with increased HIV risk. Respondents from Bagalkot were least likely to be positive for syphilis (AOR: 0.4; 95%CI: 0.2-1.0, p<.05).

**Table 1:** Adjusted odds ratios (AOR) and 95% confidence intervals (95%CI) from multivariable logistic regression models, socio-demographic, sexual behaviour and sex-work related characteristics associated with pathogen prevalence, Karnataka, South India^a^

		HIV		HSV-2		SYPHILIS		CT/NG	
		AOR	95%CI	AOR	95%CI	AOR	95%CI	AOR	95%CI
Duration		1.05^***^	(1.03-1.07)	1.08^***^	(1.06-1.10)	1.03	(0.99-1.06)	0.97	(0.93-1.02)
Married									
	Not currently married	*Ref*	*--*	*Ref*	*--*	*Ref*	*--*	*Ref*	*--*
	Married	0.70	(0.43-1.16)	1.53^**^	(1.20-1.96)	1.14	(0.63-2.05)	1.54	(0.71-3.35)
District									
	Belgaum	*Ref*	*--*	*Ref*	*--*	*Ref*	*--*	*Ref*	*--*
	Bagalkot	2.93^***^	(1.68-5.10)	1.62^**^	(1.16-2.26)	0.42^*^	(0.18-0.96)	0.33	(0.05-2.25)
	Bellary	0.98	(0.48-2.02)	0.92	(0.64-1.32)	1.18	(0.57-2.61)	2.07	(0.57-7.49)
	Shimoga	0.50	(0.23-1.10)	0.79	(0.51-1.21)	0.54	(0.20-1.35)	0.92	(0.21-4.07)
	Bangalore	0.35^**^	(0.17-0.72)	0.87	(0.59-1.29)	0.87	(0.36-1.91)	3.26	(0.94-11.29)
	Mysore	0.87	(0.44-1.74)	1.01	(0.66-1.54)	0.58	(0.22-1.25)	7.43^*^	(1.52-36.25)
Occupation									
	Transport worker	*Ref*	*--*	*Ref*	*--*	*Ref*	*--*	*Ref*	*--*
	Service	2.80^**^	(1.51-5.19)	1.11	(0.82-1.83)	2.36	(0.80-5.89)	0.76	(0.29-1.96)
	Business	1.68	(0.83-3.41)	0.96	(0.69-1.57)	1.69	(0.53-4.67)	1.04	(0.43-2.50)
	Other	1.36	(0.81-2.29)	0.89	(0.71-1.22)	2.23	(0.96-4.72)	0.78	(0.35-1.72)
Place of solicitation									
	Public place	*Ref*	*--*	*Ref*	*--*	*Ref*	*--*	*Ref*	*--*
	Brothel	2.41^**^	(1.23-4.72)	1.43	(0.89-2.21)	1.11	(0.66-3.36)	2.29	(0.73-7.01)
	Home	0.98	(0.64-1.51)	1.10	(0.84-1.50)	1.09	(0.54-1.69)	2.36	(0.86-6.48)
	Lodge	0.15	(0.02-1.14)	0.77	(0.48-1.24)	0.15	(0.16-1.84)	6.28^*^	(1.92-20.55)
Number of FSWs (6 months)									
	1	*Ref*	*--*	*Ref*	*--*	*Ref*	*--*	*Ref*	*--*
	2-4	0.96	(0.60-1.51)	0.96	(0.71-1.30)	0.92	(0.53-2.29)	0.77	(0.26-2.23)
	5+	1.00	(0.59-1.69)	1.06	(0.77-1.45)	0.95	(0.51-2.30)	1.38	(0.42-4.49)

^a^FSWs: Female sex workers; CT: Chlamydia; NG: Gonorrhea; HIV: Human immunodeficiency virus; HSV-2: Herpes simplex virus, type 2

^*^p<.05; ^**^p<.01; ^***^p<.0001

Table 2 shows the results from the multivariable models examining the association between co-infection with another STI and being HIV-positive. In the adjusted analysis, being positive for HSV-2 (AOR: 10.4; 95%CI: 6.1-17.7, p<.0001) and syphilis (AOR: 2.5; 95%CI: 1.0-6.0, p<.05) were both independently associated with HIV infection. It is important to note that the direction of causality of HIV, HSV-2 and syphilis cannot be determined through the cross-sectional nature of the data.

**Table 2:** Adjusted odds ratios (AOR) and 95% confidence intervals (95%CI) from logistic regression, pathogen co-infection, socio-demographic, sexual behaviour and sex-work related characteristics associated with HIV prevalence, clients of female sex workers, Karnataka, South India^a^

		AOR	95%CI
Pathogen co-infection			
	HSV-2	10.42^***^	(6.13-17.71)
	Syphilis	2.49^*^	(1.04-5.95)
	CT/NG	0.65	(0.12-3.47)
Duration		1.01	(0.99-1.03)
married			
	Not currently married	*Ref*	*--*
	Married	0.52^*^	(0.31-0.89)
District			
	Belgaum	*Ref*	*--*
	Bagalkot	2.48^**^	(1.35-4.56)
	Bellary	1.08	(0.45-2.58)
	Shimoga	0.55	(0.24-1.25)
	Bangalore	0.40^**^	(0.18-0.87)
	Mysore	0.92	(0.42-2.01)
Occupation			
	Transport Worker	*Ref*	*--*
	Service	2.50^**^	(1.26-4.94)
	Business	1.65	(0.78-3.48)
	Other	1.40	(0.79-2.46)
Place of solicitation			
	Public place	*Ref*	*--*
	Brothel	2.10	(0.97-4.56)
	Home	0.97	(0.60-1.55)
	Lodge	0.20	(0.03-1.29)
Number of FSWs (6 months)			
	1	*Ref*	*--*
	2-4	0.99	(0.59-1.64)
	5+	0.98	(0.54-1.77)

^a^FSWs: Female sex workers; CT: Chlamydia; NG: Gonorrhea; HIV: Human immunodeficiency virus; HSV-2: Herpes simplex virus, type 2

^*^p<.05; ^**^p<.01; ^***^p<.0001

## Discussion

This cross-sectional study characterised the prevalence of HIV and STIs among a sample of clients from six districts in Karnataka state, southern India. The results demonstrated substantial heterogeneity in the prevalence of HIV and other STIs, both by district and by type of pathogen. A strong and positive relationship between HIV infection and co-infection with other pathogens was detected. These results illustrate the complexity, not only in understanding the epidemiology of HIV and its interaction with other STIs [31,32], but also in the management of HIV-infected persons [31,33].

### Concurrent epidemics

The prevalence of HIV, syphilis and HSV-2 in the current study was slightly lower than that observed in a previous study of clients across three states in southern India (Andhra Pradesh, Maharashtra and Tamil Nadu), where prevalence was 6%, 5% and 31%, respectively [21]. Although rates are highly variable, research from southern India has estimated much lower prevalence of HIV and HSV-2 among the general male population at under 1% [1,34] and 9% [35], respectively, underscoring the importance of characterising the epidemiology of HIV and STIs among clients of FSWs. Studies of client populations conducted in countries other than India have noted similarly high rates of HIV [18,36], as well as substantially higher rates of HSV-2 [21,37-39].

Alongside strong evidence demonstrating the link between HIV and HSV-2 [40,41], the association between HIV and HSV-2 has been observed in client populations from other settings [36]. For example, our results are consistent with a recent study of clients conducted in Haiti, where the authors found that HSV-2-positive clients were also at nine-fold the odds for HIV infection [36]. Ultimately, the co-mingling of HIV and HSV-2 has far-reaching implications for prevention and management of HIV [42], as well as understanding the trajectories of both epidemics.

### Duration of sex work involvement and other heterogeneous characteristics

Although a study by Subramanian et al. provided important information regarding patterns of risk behaviour which may potentially place certain subgroups of clients at higher risk of acquiring HIV and other infections [21], the *plausibility* of biological risk does not always necessarily translate into actual, or operationalised risk. That positivity for both HIV and HSV-2 in the present study was strongly associated with duration of paying for sex, and importantly, *not* associated with frequency of FSW contact, highlights the limitations inherent in cross-sectional data, as current behaviours do not necessarily correlate with behavioural patterns at the time of pathogen acquisition [43]. Without broader information on sexual structure, and the organization of sex work, a more complete understanding of *current* risk will remain elusive. Such information could include details on the distribution of FSWs relative to clients, the overlap of clients amongst FSWs, regular commercial and non-commercial partnerships, as well as social and sexual networks [44]. Longitudinal studies focussing on *sexual structure dynamics* examining issues such as partnership patterns, and the distribution and movement of both FSWs and clients into and out of localities over time may also be a potentially useful avenue for research [45], and may be especially salient with infections that cause ulcerative diseases as the presence (and recognition) of ulcers may cause shifts in behavioural patterns.

Although somewhat lower than what was reported by Subramanian et al. (8%), the fact that almost 6% of clients reported having anal sex with another man or hijra in the 6 months prior to their interview is of some interest. In sub-analyses by district (not shown), there was a degree of heterogeneity in reporting this sexual behaviour; prevalence was under 3% in Bagalkot (1%), Shimoga (1%), Belgaum (2%) and Bellary (3%), while highest in Mysore (6%) and Bangalore Urban (12%). Results were inconsistent when this behaviour was compared against pathogen prevalence (Table 2), with those reporting anal sex with a man/hijra having higher prevalences of HSV-2, syphilis and CT/NG, and lower HIV prevalence. However, given previously published reports of high risk behaviours amongst bisexual men [46,6], and as clients who report concurrent sex with both FSWs and other males/hijras have the potential to act as a bridging population to two disconnected networks, further research into this sub-population is warranted [6].

Finally, in multivariable models, clients who reported mostly soliciting FSWs in brothels were at over two-fold the odds of being HIV-positive, compared to clients who mostly solicited in public places. This finding is consistent with a study on FSWs from Karnataka, which found that brothel-based FSWs had the highest HIV prevalence, despite having high rates of condom use [15]. The authors concluded that higher client volume, decreased likelihood of attending sexual health clinics, and increased likelihood of travel to Mumbai (where HIV prevalence has been reported to be near 50% among FSWs) may have contributed to brothel-based FSWs being at higher risk of HIV. As demonstrated in this study, the prevalence of HIV among brothel-based clients may also be an important factor in understanding the risks posed to FSWs.

### Strengths and limitations

Our study possessed a number of strengths, including the integration of biological and behavioural components. We had a large sample size from several districts in southern India, which is notable, considering the hidden nature of client populations. There were a number of limitations to the study; first and foremost, although a complex and rigorous sampling technique was used to define the sampling frame, all possible clients were not included in the sampling universe, limiting the ability to generalize our results to the total client population in each district. Notably, clients who only exchanged gifts or services (instead of money) for sex were not included in the study. Therefore, not all types of clients were included. Future studies may want to explore the possibility of using alternative sampling strategies, such as respondent-driven sampling, or snowball sampling. However, since clients represent a loosely-defined, highly heterogeneous and disconnected group, the application of these alternative strategies may prove challenging. Expanding the definition of clients to include those that exchange sex for non-monetary transactions should be explored in future studies. Second, all responses were self-reported, and the possibility of social desirability bias cannot be discounted. Third, data were cross-sectional, and thus causality could not be inferred from our study.

## Conclusions

In conclusion, this study presented a comprehensive description of HIV and other STIs among clients from six districts in southern India. Our results demonstrate a remarkable degree of heterogeneity, with respect to pathogen prevalence within this hidden and important bridging population. Importantly, HIV and HSV-2 were highly associated with a longer duration of involvement with sex workers, and this finding, along with the association found between infection with HIV, HSV-2 and syphilis highlights the complexity in understanding the exact determinants of pathogen prevalence. As noted by Lowndes et al. [19,20], targeting of clients by intervention programs is a necessary component of a comprehensive response to HIV. Thus, this study can serve as a useful platform to inform present initiatives, as well as future research into client-FSW partnerships and interactions, and how they evolve over time. Given the higher rates of HIV in clients that solicit sex work in brothels, aligning with previously published work on the higher risk of HIV amongst brothel-based FSWs, improved strategies to engage with both clients and FSWs in brothels may be essential. At the same time, because present behaviours do not necessarily correlate with behaviours at the time of pathogen acquisition, more research is needed in understanding the trajectory of clients in seeking sex workers. A more nuanced understanding of when risk shifts from *acquisition* of HIV and other pathogens, to *transmission* to sex partners would likely inform the design, timing, and placement of effective and broad-based intervention programming.

### List of abbreviations used

HIV: human immunodeficiency virus; STI: sexually transmitted infections; FSW: female sex workers; HSV2: herpes simplex virus type 2; CT: Chlamydia; NG: gonorrhoea; SE: standard error; AOR: adjusted odds ratios; 95% CI: 95% confidence intervals; MSM: men who have sex with men; VIF: variance inflation factor.

## Competing interests

The authors declare they have no competing interests.

## Authors’ contributions

SYS was responsible for conducting the analysis, interpretation of results and writing of the manuscript; KND made substantial contributions to data analysis and interpretation and revised the manuscript critically and made important intellectual contributions to the manuscript; SRP, SI, and BMR contributed to the project’s conception, design, implementation and progress, and provided extensive feedback and edits; SM and JFB were extensively involved in the conception, methodology, and organization of the project, and provided extensive intellectual guidance and substantial feedback for the manuscript.

## Supplementary Material

Additional file 1 Distribution of socio-demographic, sexual behaviour and sex-work related characteristics, clients of female sex worker, Karnataka, South India Click here for file

Additional file 2 Pathogen prevalence, by socio-demographic, sexual behaviour and sex-work related characteristics, clients of female sex workers, Karnataka, South India. Click here for file
