# Supplementary material for: Prevalence of HIV and sexually transmitted infections among clients of female sex workers in Karnataka, India: a cross-sectional study
Source: BMC Public Health. 2011 Dec 29;11(Suppl 6):S4. doi: 10.1186/1471-2458-11-S6-S4 (PMC3287557; doi:10.1186/1471-2458-11-S6-S4)
Supplement: Additional file 1 — Distribution of socio-demographic, sexual behaviour and sex-work related characteristics, clients of female sex worker, Karnataka, South India [file 1471-2458-11-S6-S4-S1.pdf]

Supplemental Table 1: Distribution of socio-demographic, sexual behaviour and sex-work related characteristics, clients of female sex workers, Karnataka, South India (N=2,757)[a]

	Characteristic		%
Socio-demographic	Age		
		18-24	29.7
		25-29	24.6
		30-39	28.7
		40+	17.0
		Mean	*30.4*
	Marital status		
		Married	62.1
		Separated	2.6
		Never married	35.3
	Occupation		
		Transport	25.9
		Service	13.8
		Business	11.6
		Other (including Labourer)	48.7
	Can read and write		
		No	25.3
		Yes	74.7
	Districts		
		Belgaum	17.3
		Bagalkot	17.1
		Bellary	14.2
		Shimoga	15.5
		Bangalore	30.6
		Mysore	5.4
Sexual Behaviour	Age at 1st sex		
		<18	15.4
		18-20	52.9
		21-24	21.1
		25+	10.6
		Mean	*20.0*
	Have an intimate partner		66.2
	Condom use in last sex with intimate partner		10.3
	Never use condoms with intimate partner		86.5
	Anal sex with intimate partners		4.6
	Anal sex with MSM/hijra (6 months)		5.5

Sex-Work Related	Age at 1st paid sex	
	<18	7.4
	18-20	47.2
	21-24	26.7
	25+	18.7
	Mean	*21.5*
	Duration of paid sex	
	1 year or less	14.3
	2-4	25.3
	5-9	22.4
	10+	38.0
	Mean	*8.9*
	Place of solicitation, FSWs	
	Public places	36.0
	Brothel	9.8
	Home	47.8
	Lodge	6.4
	Number of FSWs (Total: 6 months)	
	1	17.0
	2-3	40.7
	4-5	22.2
	6-9	12.6
	10+	7.4
	Mean	*4.3*
	Number of Occasional FSWs (6 months)	
	0	14.3
	1	12.9
	2-4	51.8
	5+	21.1
	Mean	*3.4*
	Number of Regular FSWs (6 months)	
	0	57.8
	1	28.5
	2-4	10.4
	5+	3.4
	Mean	*0.9*
	Condom use in last sex, occasional FSW	66.9
	Never use condoms, occasional FSW	26.3
	Condom use in last sex, regular FSW	59.9
	Never use condoms, regular FSW	32.4
	Ever asked for anal intercourse, FSW	11.2

	Ever had anal sex, FSW	7.8
	Condom use in last anal sex, FSW	57.1
Pathogen Prevalence	CT	1.8
	NG	0.5
	CT/NG	2.2
	Active syphilis	3.6
	HIV	5.6
	HSV-2	28.4

[a]MSM: Men who have sex with men; FSW: Female sex workers; CT: Chlamydia; NG: Gonorrhea; HIV: Human immunodeficiency virus; HSV-2: Herpes simplex virus, type 2
