# Supplementary material for: Prevalence of HIV and sexually transmitted infections among clients of female sex workers in Karnataka, India: a cross-sectional study
Source: BMC Public Health. 2011 Dec 29;11(Suppl 6):S4. doi: 10.1186/1471-2458-11-S6-S4 (PMC3287557; doi:10.1186/1471-2458-11-S6-S4)
Supplement: Additional file 2 — Pathogen prevalence, by socio-demographic, sexual behaviour and sex-work related characteristics, clients of female sex workers, Karnataka, South India. [file 1471-2458-11-S6-S4-S2.pdf]

Supplemental Table 2: Pathogen prevalence, by socio-demographic, sexual behaviour and sex-work related characteristics, clients of female sex workers, Karnataka, South India[a]

	HIV % (N=2,745)	p	HSV-2 % (N=2,610)	p	Syphilis % (N=2,613)	p	CT/NG % (N=2,736)	p
Prevalence	**5.6**		**28.4**		**3.6**		**2.2**	
Age		.002		<.0001		.044		.069
18-24	2.7		11.6		1.9		2.2	
25-29	5.6		22.3		3.3		3.7	
30-39	7.8		38.9		4.7		1.5	
40+	7.2		48.7		4.9		1.3	
Mean	*32.7*		*34.6*		*33.4*		*28.2*	
Marital status		<.0001		<.0001		.033		.505
Married	5.7		35.3		4.0		2.2	
Separated	25.5		65.1		8.5		0.0	
Never married	4.2		13.7		2.4		2.5	
Occupation		.007		.267		.202		.591
Transport	3.3		25.0		1.9		2.8	
Service	8.6		30.6		4.7		1.9	
Business	5.7		31.8		3.4		2.7	
Other	6.0		28.6		4.2		1.9	
Can read and write		<.0001		<.0001		.0006		.019
No	9.6		36.7		6.2		1.0	
Yes	4.3		25.5		2.7		2.6	
Districts		<.0001		.055		.122		.005
Belgaum	6.1		27.7		4.3		1.4	
Bagalkot	13.5		36.3		1.9		0.5	
Bellary	6.1		26.5		5.5		2.4	
Shimoga	2.6		24.9		2.3		1.2	
Bangalore	2.4		26.5		3.9		3.6	
Mysore	5.4		31.3		2.9		5.0	
Age at 1st sex		.027		.488		.492		.079
<18	4.0		29.4		2.7		3.2	
18-20	6.9		28.3		3.5		2.6	
21-24	3.7		26.1		3.6		1.3	
25+	5.5		31.5		5.2		0.9	
Mean	*20.0*		*20.1*		*20.5*		*19.1*	
Have an intimate partner		.186		<.0001		.251		.254
No	4.7		18.2		2.9		2.7	
Yes	6.1		33.5		3.9		2.0	
Condom use in last sex with intimate partner		.619		.041		.939		.008
No	6.0		34.5		4.0		1.6	
Yes	7.2		25.2		3.8		4.9	

Use condoms with intimate partner		.627		.072		.968		.229
Sometimes use	6.9		28.0		3.9		3.1	
Never use	5.9		34.3		3.9		1.8	
Anal sex with intimate partners		.057		.860		.562		.941
No	5.8		31.7		3.9		1.9	
Yes	1.1		33.7		2.5		1.7	
Anal sex with MSM/hijra (6 months)		.338		.186		.765		.012
No	5.7		27.8		3.5		2.0	
Yes	4.1		36.8		4.0		6.2	
Age at 1st paid sex		.055		.307		.421		.398
<18	2.7		27.8		2.1		3.7	
18-20	6.6		27.0		3.2		2.4	
21-24	3.9		28.0		3.7		2.1	
25+	6.9		32.4		4.9		1.5	
Mean	*21.8*		*22.0*		*22.3*		*20.5*	
Duration of paid sex		.001		<.0001		.347		.503
1 year or less	2.1		10.5		1.8		1.9	
2-4	4.5		18.3		3.2		2.8	
5-9	6.1		25.1		4.2		2.6	
10+	7.5		43.8		4.2		1.8	
Mean	*10.8*		*12.6*		*11.1*		*7.7*	
Place of solicitation		.003		.142		.179		.026
Public places	4.6		27.1		3.8		1.7	
Brothel	9.4		31.1		6.0		2.8	
Home	6.3		29.8		3.1		1.9	
Lodge	0.6		19.2		1.9		6.5	
Number of FSWs (6 months)		.617		.091		.578		.416
1	6.7		27.4		2.9		1.8	
2-3	4.7		27.0		3.5		2.1	
4-5	6.2		28.1		3.5		1.9	
6-9	5.7		28.0		5.3		4.1	
10+	6.6		38.7		3.0		2.1	
Mean	*4.7*		*4.7*		*4.2*		*4.8*	
Number of Occasional FSWs (6 months)		.165		.068		.484		.331
0	8.1		33.5		3.4		2.3	
1	4.3		23.9		2.0		1.6	
2-4	5.1		27.0		3.7		1.9	
5+	6.1		30.8		4.2		3.3	
Mean	*3.7*		*3.7*		*3.4*		*4.0*	

Number of Regular FSWs (6 months)		.011		.054		.887		.911
0	4.6		26.4		3.5		2.2	
1	8.0		30.1		3.8		2.1	
2-4	5.8		30.8		2.9		2.7	
5+	3.8		37.6		4.0		1.9	
Mean	*1.0*		*1.0*		*0.8*		*0.8*	
Condom use in last sex, occasional FSW		.579		.017		.751		.173
No	4.9		31.1		3.4		1.4	
Yes	5.4		25.6		3.7		2.6	
Use condoms, occasional FSW		.478		.003		.624		.537
Sometimes use	5.4		25.5		3.7		2.4	
Never use	4.7		32.8		2.3		1.8	
Condom use in last sex, regular FSW		.386		.139		.931		.276
No	6.3		34.2		3.8		1.6	
Yes	7.6		29.1		3.9		2.8	
Never use condoms, regular FSW		.348		.039		.797		.673
Sometimes use	7.4		28.9		3.7		2.5	
Never use	6.5		35.8		4.1		2.0	
Ever asked for anal intercourse, FSW		.187		.737		.247		.660
No	5.9		28.1		3.7		2.2	
Yes	3.5		30.3		2.4		2.7	
Ever had anal sex, FSW		.082		.997		.745		.428
No	5.9		28.3		3.6		2.1	
Yes	2.7		28.3		3.1		3.4	
Condom use in last anal sex, FSW		.327		.109		.423		.587
No	1.3		20.2		1.8		4.0	
Yes	3.7		34.7		4.2		2.9	
CT		.495		.492		.817	n/a	--
Negative	5.7		28.3		3.6			
Positive	3.6		32.9		3.0			
NG		.129		.0001		<.0001	n/a	--
Negative	5.6		28.1		3.4			
Positive	7.9		73.2		34.6			
CT/NG		.767		.027		.054	n/a	--
Negative	5.7		28.0		3.4			
Positive	4.7		42.3		10.0			

Active syphilis			<.0001		<.0001	n/a	--		.054
	Negative	5.2		26.9				2.2	
	Positive	21.3		68.0				6.5	
HSV-2			<.0001	n/a	--		<.0001		.027
	Negative	1.7				1.6		1.9	
	Positive	16.2				8.6		3.5	
HIV		n/a	--		<.0001		<.0001		.767
	Negative			25.2		3.0		2.2	
	Positive			78.9		13.1		1.8	

[a]MSM: Men who have sex with men; FSW: Female sex workers; CT: Chlamydia; NG: Gonorrhea; HIV: Human immunodeficiency virus; HSV-2: Herpes simplex virus, type 2
